# Giant intraperitoneal non-pancreatic pseudocyst: a case report

**DOI:** 10.1186/s13256-024-04503-5

**Published:** 2024-04-29

**Authors:** Yohannis Derbew Molla, Aklilu Yiheyis Abraha, Yilkal Ademe Belay, Bewketu Abebe Alemu, Hirut Tesfahun Alemu, Samuel Addisu Abera

**Affiliations:** 1 Department of Surgery, College of Medicine and Health Sciences, Gondar, Ethiopia; 2 Department of Pathology, College of Medicine and Health Sciences, Gondar, Ethiopia; 3 University of Gondar, Gondar, Ethiopia

**Keywords:** Non-pancreatic, Pseudocyst, Bladder, Inflammatory, Case report

## Abstract

**Introduction:**

Non-pancreatic pseudocysts are rare lesions that typically form from the omentum and mesentery. These cysts have a thick fibrotic wall made up of fibrous tissue and may show signs of calcifications and inflammatory changes. The fluid inside them can vary, ranging from hemorrhage and pus to serous or sometimes chylous content. In most cases, these cysts appear as a result of trauma, surgery, or infection.

**Case presentation:**

A 35-year-old male patient from Ethiopia presented with swelling in his lower abdomen that had been present for 2 years. Initially, the swelling was small but gradually increased in size. The patient experienced frequent urination but no pain or difficulty during urination, urgency, intermittent urination, or blood in the urine. The swelling was initially painless but became painful 2 months prior to his presentation. Abdominal computed tomography scans revealed a well-defined, lobulated peritoneal lesion measuring 16 × 12 × 10 cm, consisting primarily of fluid-filled cysts with a thick, enhancing wall and septa. Additionally, there was a large, heterogeneous enhancing soft tissue component measuring 8 × 6 cm. As a result, the cystic mass was surgically removed in its entirety with partial removal of the bladder wall, and the patient was discharged in an improved condition.

**Conclusion:**

Primary non-pancreatic pseudocysts are extremely rare lesions that must be differentiated from other possible causes of cystic lesions within the peritoneal or retroperitoneal regions. Surgeons should be aware of the potential occurrence of these lesions, which may have an unknown origin.

**Supplementary Information:**

The online version contains supplementary material available at 10.1186/s13256-024-04503-5.

## Background

Non-pancreatic pseudocysts are rare and intriguing lesions that usually arise from the mesentery and omentum. These captivating lesions are believed to be the liquefied remains of an abscess or hematoma that did not completely heal [1]. Nonpancreatic pseudocysts have been discovered after surgical procedures such as ventriculoperitoneal shunts, major pelvic operations in premenopausal women with visible ovaries within the cysts, and intraperitoneal dialysis catheters—especially after an infection [2]. The signs can be quite ambiguous, with the presence of a palpable and easily movable abdominal mass during examination. Those suffering from nonpancreatic pseudocysts might experience intense abdominal discomfort if infection or bleeding occurs within the pseudocyst. The presence of a large pseudocyst can lead to sensations of stomach fullness, nausea, and vomiting as well as exerting pressure on surrounding tissues such as the intestine [3]. Ultrasonography often detects a hypoechoic mass filled with echogenic material, while computed tomography (CT) and magnetic resonance (MR) images reveal a thick-walled cystic tumor with fluid–fluid levels indicating hemorrhagic or purulent contents [4]. Surgery is the most important and commonly used method for treating pseudocysts. It involves completely removing the cyst to minimize the chances of recurrence. Carefully separating the cyst from the adhering bowel and retroperitoneal tissues is necessary [5]. In this report, we bring forth a compelling case involving an exceptionally large nonpancreatic pseudocyst, which is suspected to have originated from the bladder.

## Case presentation

A 35-year-old Ethiopian male patient presented with swelling in his lower abdomen that had been present for 2 years. The swelling started off small but gradually increased in size over time. Along with the swelling, he experienced frequent urination, but without any pain or discomfort during urination, urgency, intermittent urination, or blood in the urine. A total of 2 months prior to his presentation, the swelling became painful. The patient had no history of previous abdominal trauma or surgery, and had never been treated for tuberculosis. He denied any loss of appetite, fever, weight loss, or night sweats. He had no known allergies and no history of constipation, blood in the stool, or black, tarry stools. There was no swelling in any other part of his body.

**Fig. 1:**
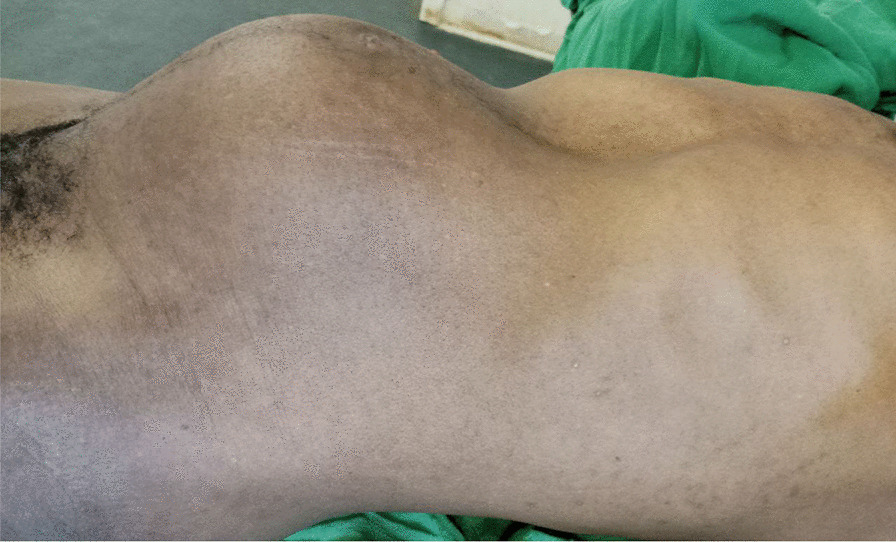
The external appearance abdominopelvic mass

On abdominal examination, there was a 20 × 13 cm non-tender abdominopelvic mass that moved laterally but not vertically (Fig. 1). It was firm in consistency and had no attachment to the overlying skin or underlying tissue. Otherwise, there was no organomegaly or sign of fluid collection. The per-rectal examination was unremarkable.

**Fig. 2:**
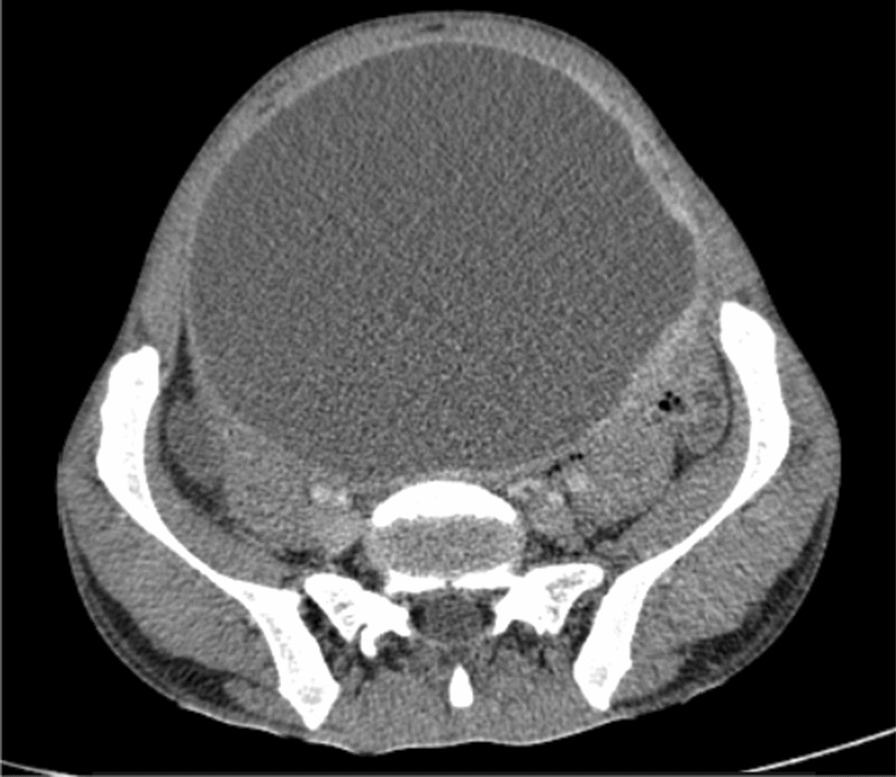
Axial abdominal CT of the patient showing cystic mass

**Fig. 3:**
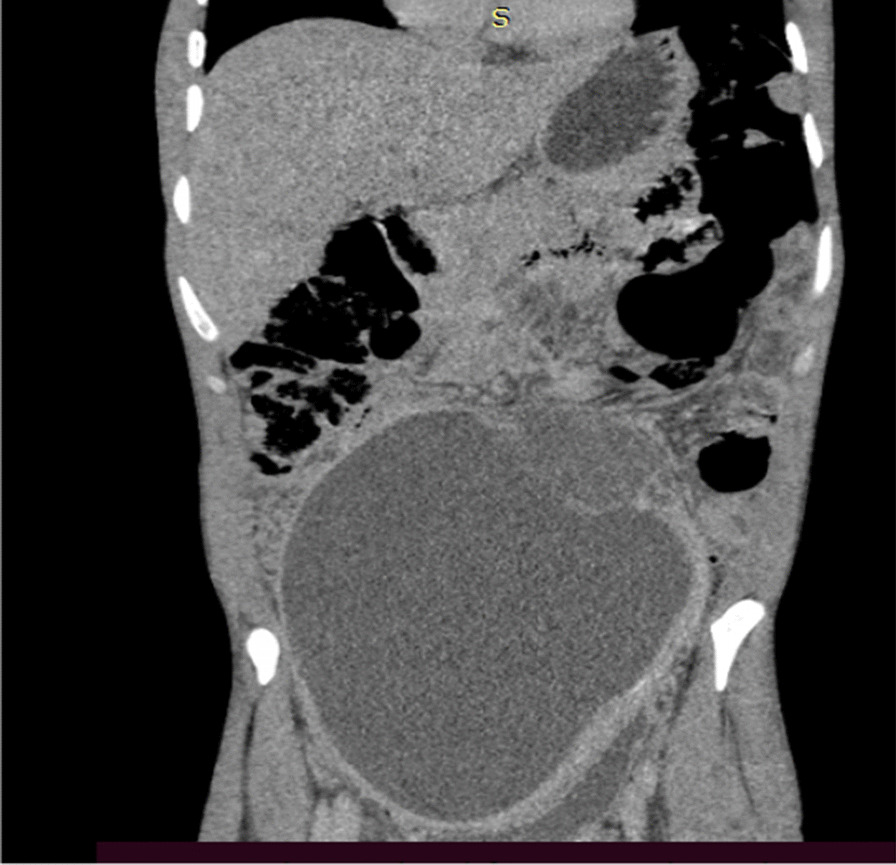
CT scan showing a predominantly cystic lesion that had an enhancing wall with heterogeneous mural soft tissue

**Fig. 4:**
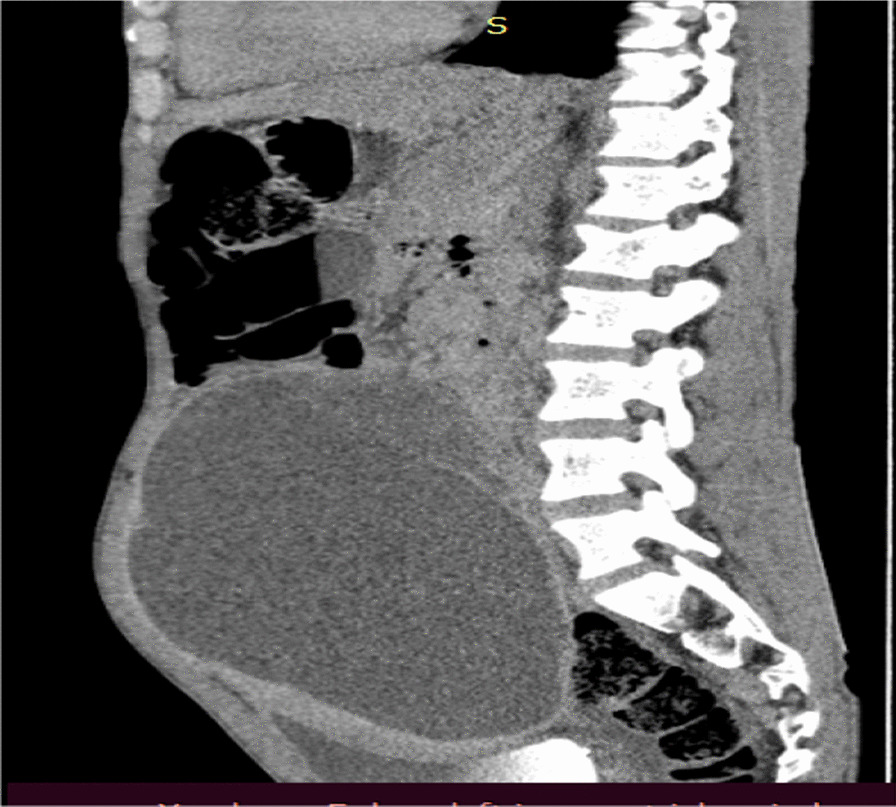
Sagittal view of the abdominal CT scan of the patient

Blood examinations were all normal. On imaging, abdominal ultrasound (US) showed a 20 × 13 × 16 cm predominantly cystic huge intra-abdominal mass with soft tissue around the left superolateral aspect, and there was moderate hydronephrosis on the right side, likely due to the mass effect. Subsequent pre- and post-contrast abdominal CT scans showed a 16 × 12 × 10 cm well-defined lobulated predominantly cystic peritoneal lesion with a thick enhancing wall and septa and a large heterogeneous enhancing soft tissue mural component measuring 8 × 6 cm (Figs. 2, 3, 4). The mass extended inferiorly into the pelvis, causing the urinary bladder to be pushed anteriorly toward the left side. The mass also exerted pressure on the descending and ascending colon, as well as the major abdominal blood vessels, pushing them posteriorly. The small bowel loops were displaced laterally, suggesting that the mass originated from the peritoneum. The other abdominal organs appeared normal, and there were no signs of ascites or enlarged lymph nodes. The mass also compressed the right proximal ureter, resulting in moderate dilation of the pelvi-caliceal system. However, no renal stone was observed.

**Fig. 5:**
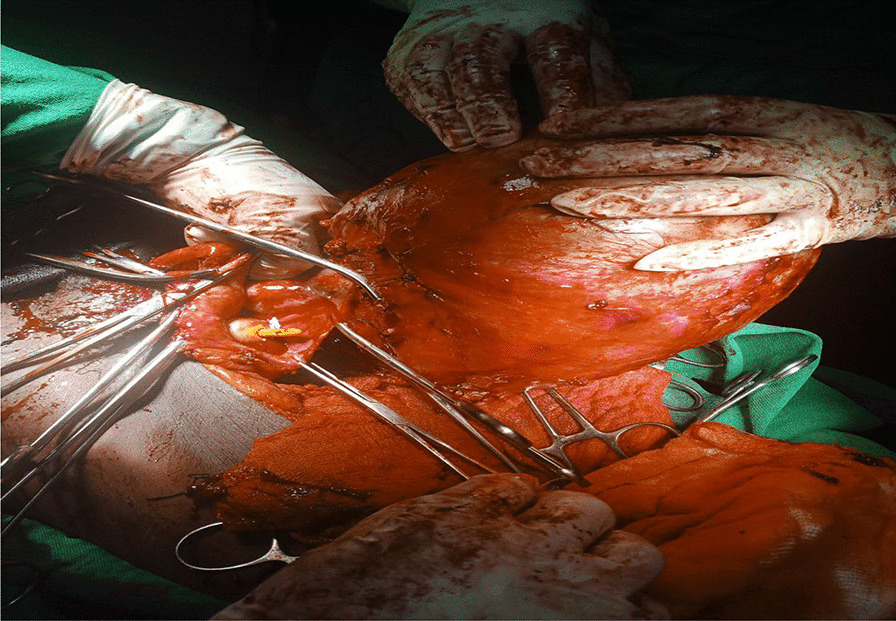
Intraoperative picture showing the mass arising from the superoposterior aspect of the bladder

**Fig. 6:**
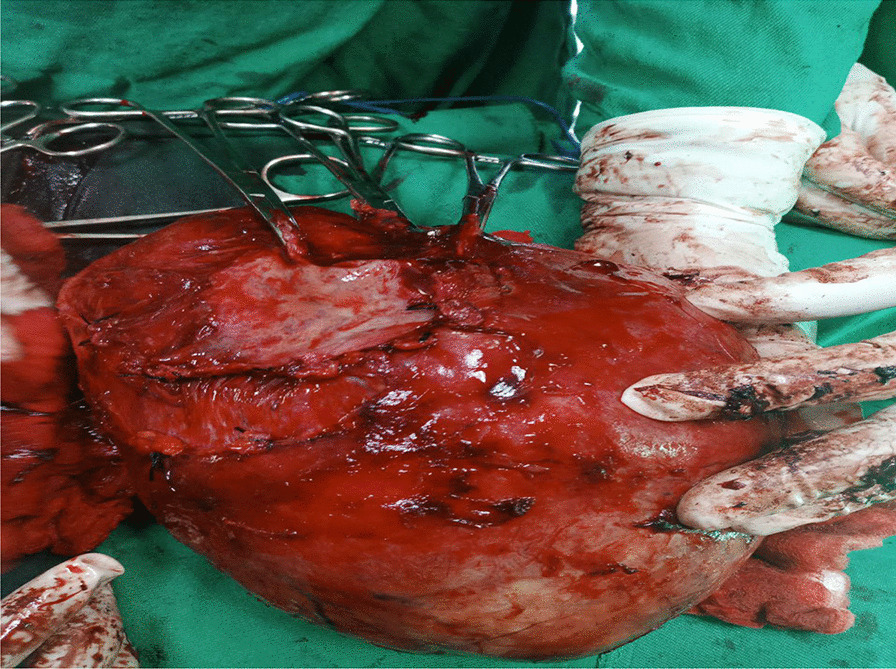
The gross appearance of the mass after total excision with partial cystectomy

**Fig. 7:**
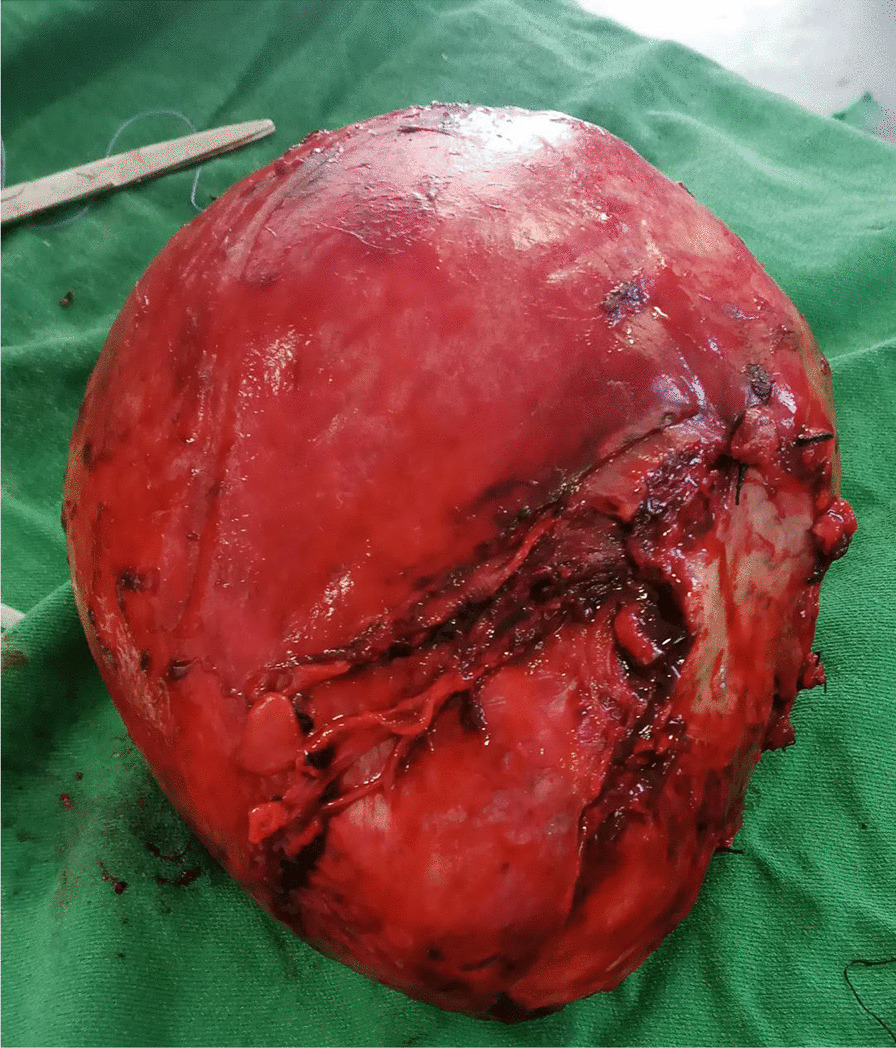
The gross appearance of the cystic mass (2.8 kg in weight)

**Fig. 8:**
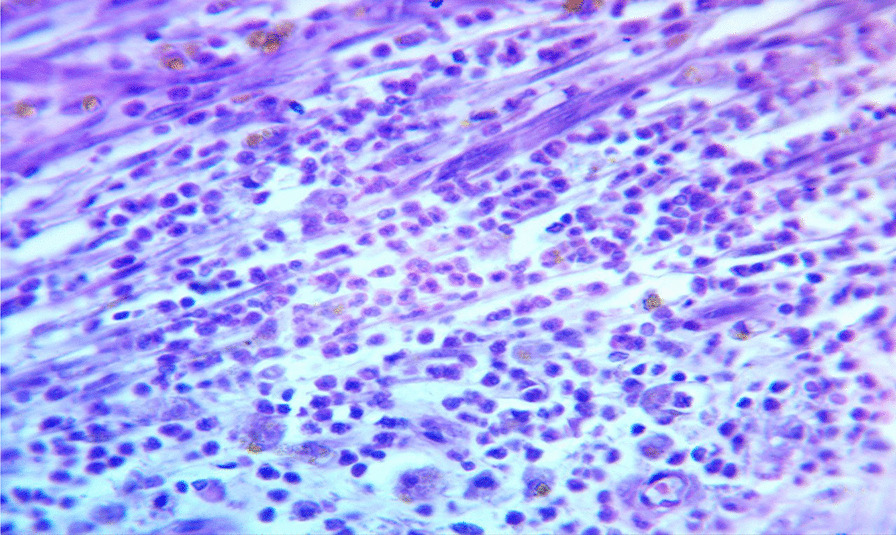
Lymphoplasmacytic inflammatory cell infiltrate and hemosiderin laden macrophages

**Fig. 9:**
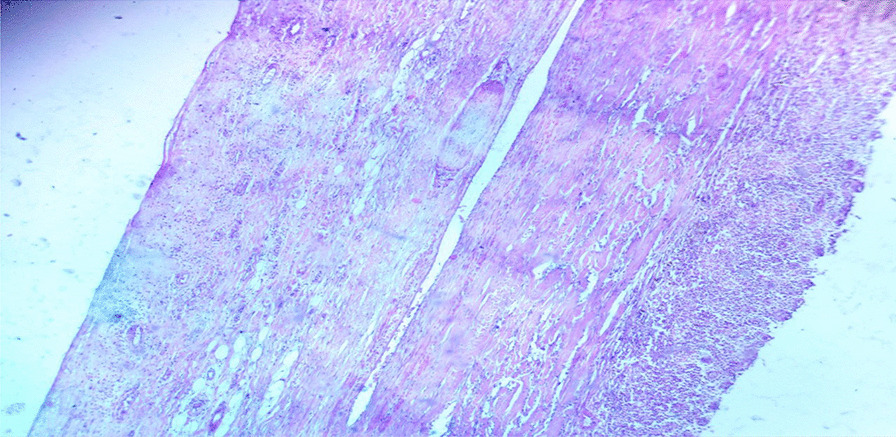
Histopathology showing fibrosis and smooth muscle bundles with no epithelium or features of malignancy

The patient underwent surgery to remove a large cystic mass in the abdominopelvic area. During the operation, it was discovered that the mass was a 20 × 10 × 10 cm globular cystic mass attached to the back and top of the urinary bladder (Fig. 5). The mass was slightly adhered to the abdominal wall and omentum, but there was a clear separation between the mass and the mesentery. No other organs or bowels showed any abnormalities. The entire cystic mass was removed along with a partial removal of the bladder (Figs. 6, 7). The bladder was repaired in two layers, and the abdomen was closed. The surgery was performed by a senior general surgeon and general surgery residents. After the surgery, the patient received ceftriaxone and tramadol intravenously for pain management. Wound care was provided daily. A urinary catheter was kept in place for 7 days and then removed. The patient recovered well and was discharged without any complications. Histopathologic analysis of the removed mass showed a fibrovascular wall, amorphous secretory materials, lymphoplasmacytic inflammatory cell infiltrates, macrophages containing hemosiderin due to bleeding within the cyst, fibrosis, and smooth muscle bundles. No signs of malignancy or abnormal epithelial cells were found (Figs. 8, 9 and Additional file 1).

## Discussion

Non-pancreatic pseudocysts are uncommon lesions that typically develop in the mesentery and omentum. These cysts have a thick, fibrous wall that may become calcified and undergo inflammatory changes. They often contain blood, pus, serous fluid, or chylous fluid. Non-pancreatic pseudocysts are usually caused by trauma, surgery, or infections [1]. There have been reports of non-pancreatic pseudocysts occurring after certain surgeries, such as those involving ventriculoperitoneal shunts and intraperitoneal dialysis catheters, especially when there is an infection. They have also been seen in premenopausal females who have undergone major pelvic surgery, with the cysts containing the ovaries [2]. Pseudocysts are cystic masses without an inner cellular lining, and they are unrelated to pancreatitis [4]. Unlike true cysts, pseudocysts do not have an epithelial lining. While pancreatic pseudocysts are commonly associated with acute pancreatitis, non-pancreatic pseudocysts do not have high levels of amylase or lipase in the fluid inside them [6, 7].

Mesenteric cysts are a diverse group of cysts that can be congenital or acquired and originate from various tissues in the abdomen. The term “mesenteric cyst” refers to the location of the cyst rather than its specific histological diagnosis. These cysts can arise from lymphatic vessels, mesothelial cells, gastrointestinal tissues, or be pseudocysts [8]. The most common locations for mesenteric cysts are the small bowel mesentery and large bowel mesentery, particularly in the right colon. In rare cases, they have been found in the mesentery of the descending colon, sigmoid, or rectum. Mesenteric cysts can vary in size, with giant mesenteric cysts being those larger than 10 cm [9].

Patients with nonpancreatic pseudocysts may not have any specific symptoms, but they may have an easily palpable and movable abdominal mass. In cases where there is infection or bleeding within the pseudocyst, sudden abdominal pain may occur. Larger pseudocysts can exert pressure on nearby structures, leading to symptoms such as feeling full in the stomach, vomiting, and constipation [3]. Commonly, patients present with nonspecific abdominal pain, bloating, pain radiating to the legs, swelling in the lower limbs, weight loss, and fever [5].

To diagnose pseudocysts, abdominal CT scanning and ultrasonography are used. Pseudocysts typically have thick walls and are fluid-filled cysts that can be either unilocular or multilocular. Over time, the cysts may calcify, giving them the appearance of an eggshell. Histologically, the cyst wall is composed of fibrous tissue without an epithelial lining [3]. In an ultrasound, non-pancreatic pseudocysts show echogenic material within the cyst and thick walls. On CT and MRI scans, they appear as cystic masses with thick walls, which may contain fluid with hemorrhagic, pus, or chylous content. MRI can clearly visualize the fatty content of the cyst using frequency selective fat saturation or chemical shift imaging [1]. The typical findings on CT imaging of a nonpancreatic pseudocyst include a thick-walled cystic mass that may contain a fluid–fluid level. The portion of the cyst that is lower in position shows a density similar to water, while the upper portion shows a density similar to fat. These two elements can easily mix with the patient’s movement, resulting in the formation of a fluid–fluid level in approximately 30 minutes [8]. CT scans also provide important information about the location, size, and shape of the lesion, as well as any involvement of nearby structures [10]. Pseudocysts are cystic tumors that lack a cellular lining. Pathologically, they are characterized by septated cystic masses with thick walls that typically contain hemorrhagic or purulent (pus-like) contents [4].

When considering the differential diagnoses for pseudocysts, it is important to differentiate them from both benign and malignant cystic lesions. In this case, the patient’s lack of constitutional symptoms, normal tumor markers, and the presence of a well-defined cyst on CT scan that does not appear to invade surrounding structures led us to consider it a benign cyst. One possible alternative diagnosis was an echinococcal cyst, caused by an infectious disease called echinococcosis. However, this was ruled out owing to the absence of daughter cysts and normal echinococcal titers. Neoplastic cystic lesions were also considered but deemed less likely, as the cystic mass had a well-defined wall without any signs of invasion into surrounding structures. This was later confirmed by histopathologic examinations. However, based on the above findings, it was not possible to completely exclude the possibility of malignancy. Therefore, we decided to perform an exploratory laparotomy of the mass to establish a definitive diagnosis [11].

The recommended treatment for nonpancreatic pseudocysts is total surgical excision. This can be achieved through enucleation using blunt or sharp dissection, segmental bowel resection, or partial cystectomy. Other options such as debridement, marsupialization, or partial excision are considered inadequate due to concerns about malignant cell seeding, septic complications, and the risk of recurrence. These concerns mainly arise from diagnostic uncertainty, although there is no evidence of such occurrences in nonpancreatic pseudocysts. If diagnostic uncertainty were not an issue, nonpancreatic pseudocysts could be treated similarly to pancreatic pseudocysts, which would likely yield satisfactory results due to their histopathological similarities. Choosing options other than total surgical excision can avoid the need for extensive resection of adjacent organs such as the bowel, bladder, or parenchyma [12, 13].

During the excision of the pseudocyst, it is crucial to remove the cyst completely to prevent recurrence caused by residual cyst wall. Careful removal from adjacent bowel or retroperitoneal organs is necessary. In the case of large cysts, fluid can be aspirated before continuing with the dissection. Laparoscopic excision is an alternative if expertise is available, as it allows for work in narrow spaces and is associated with less post-operative pain, faster recovery, and shorter hospital stays. The local recurrence rates after total excision are low [5]. Finally, the patient in this case reported being satisfied with the care provided.

## Conclusion

Primary non-pancreatic pseudocysts are extremely uncommon and must be differentiated from other cystic lesions in the abdomen or retroperitoneum. Surgeons should be aware of the potential presence of these lesions with unknown origins, and in our patient, we suspect it originated from the bladder. Surgical removal is the only way to eliminate the possibility of malignant tumors and confirm the diagnosis. The cause of primary non-pancreatic pseudocysts is still unknown. They typically do not cause symptoms until they reach a large size and begin to compress nearby structures. When dissecting large pseudocysts, caution must be exercised to avoid unintentional damage to vital structures, which could increase morbidity. The entire cyst wall must be completely excised to prevent recurrence.

### Supplementary Information

**Additional file 1.** Adjacent to Figure 8 and 9.

## Data Availability

The authors of this manuscript are willing to provide any additional information regarding the case report.

## Abbreviations

CT: Computed tomography
MRI: Magnetic resonance imaging
US: Ultrasound
