# Supplementary figures and images for: Giant intraperitoneal non-pancreatic pseudocyst: a case report
Source: J Med Case Rep. 2024 Apr 29;18:212. doi: 10.1186/s13256-024-04503-5 (PMC11057145; doi:10.1186/s13256-024-04503-5)

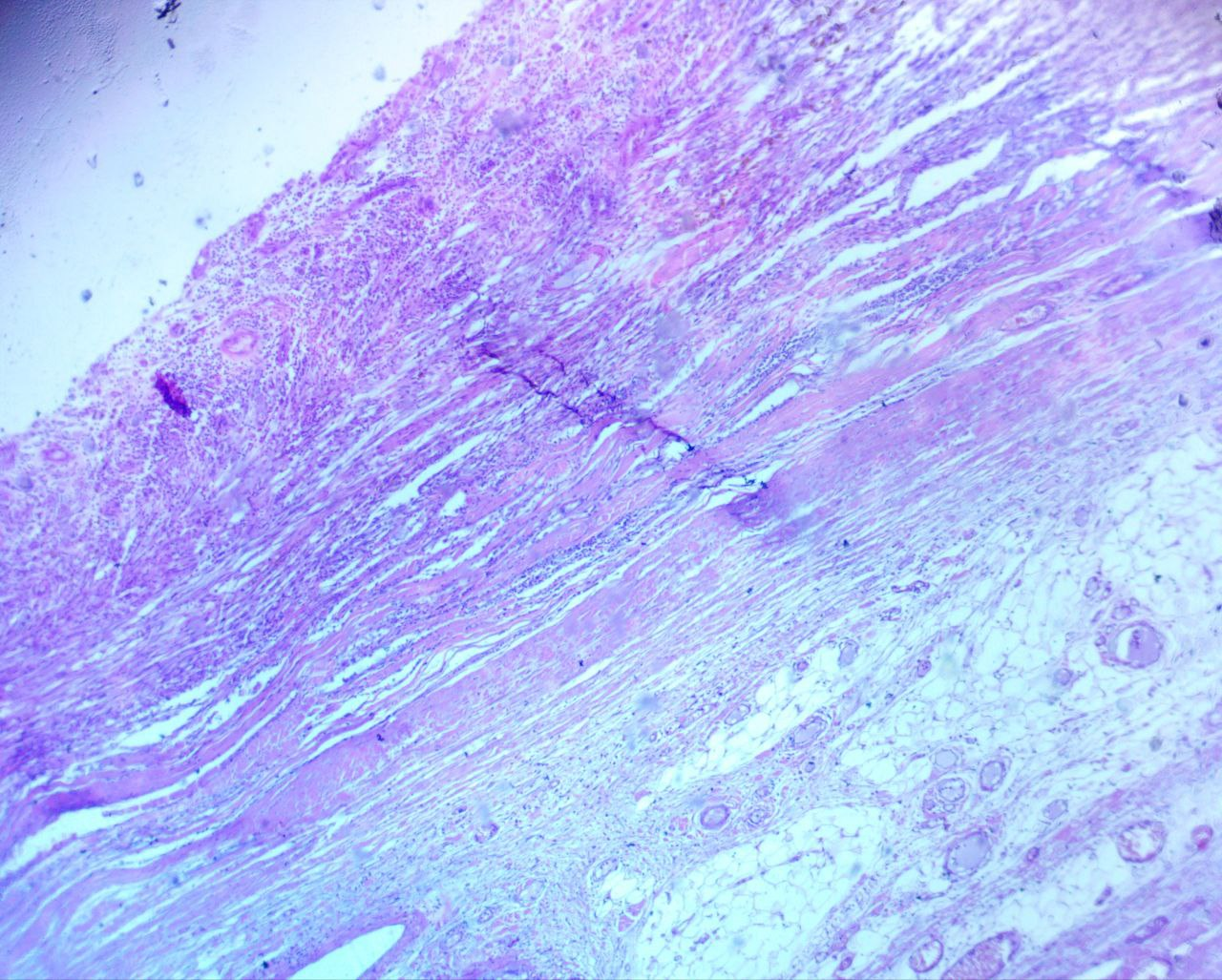

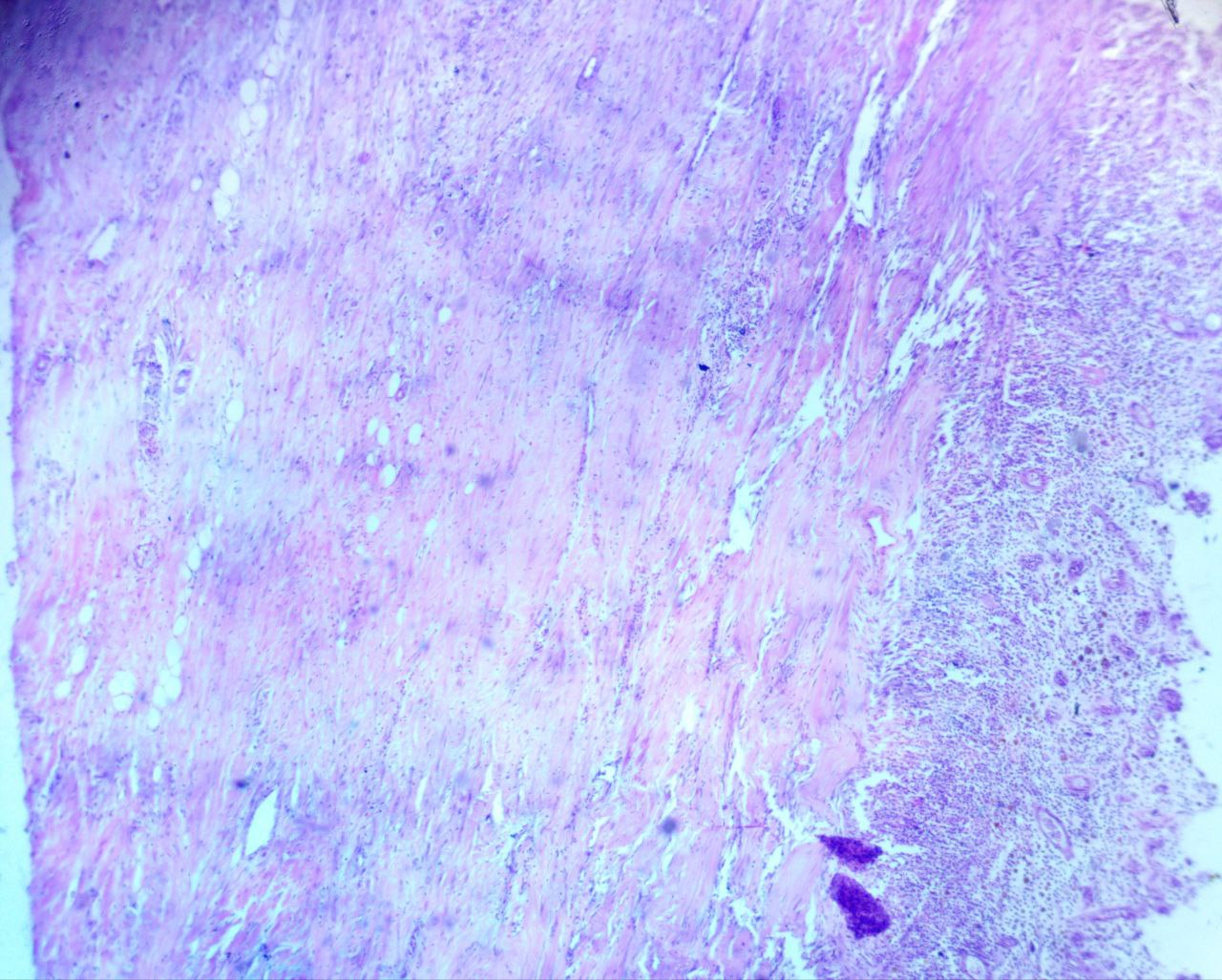

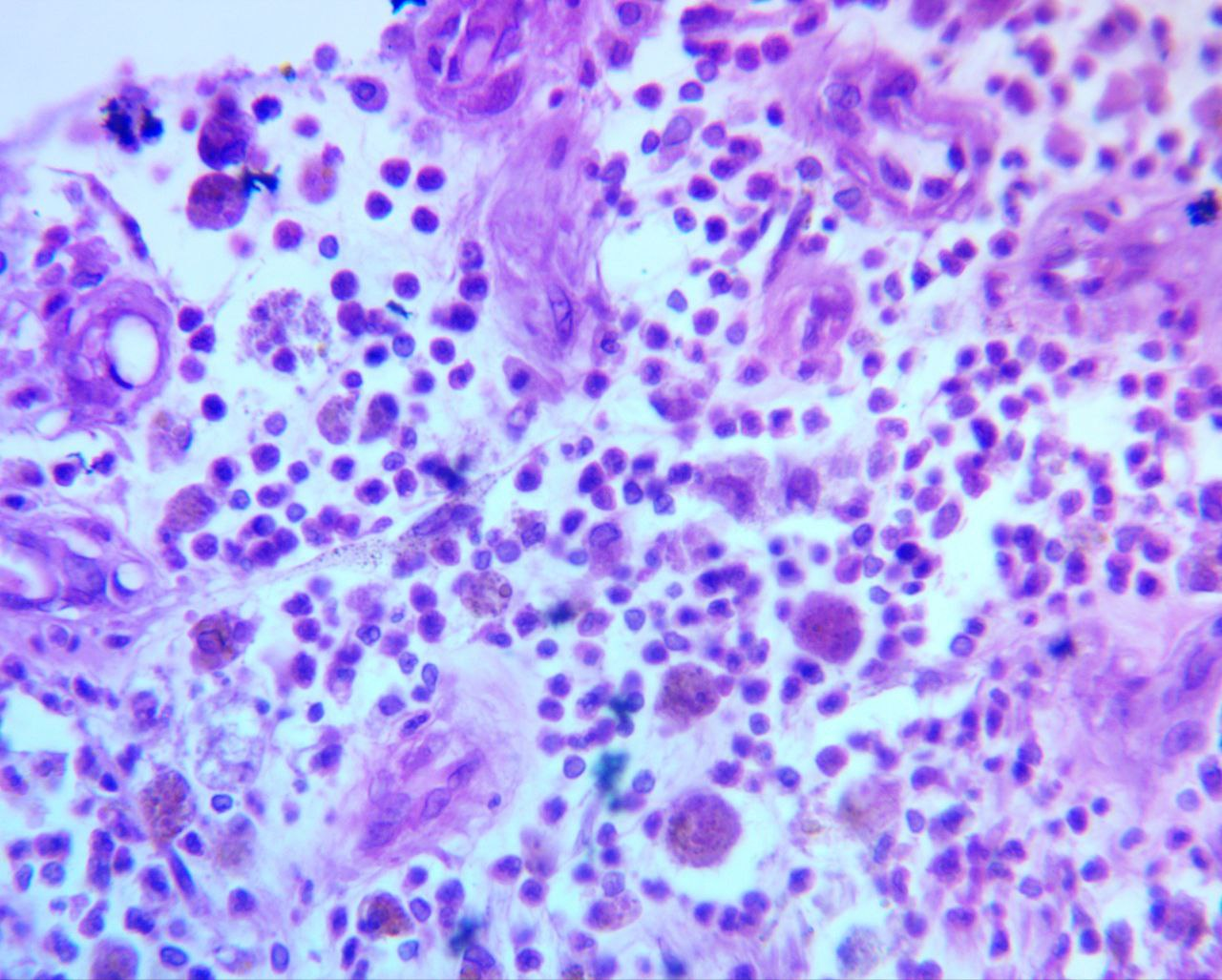

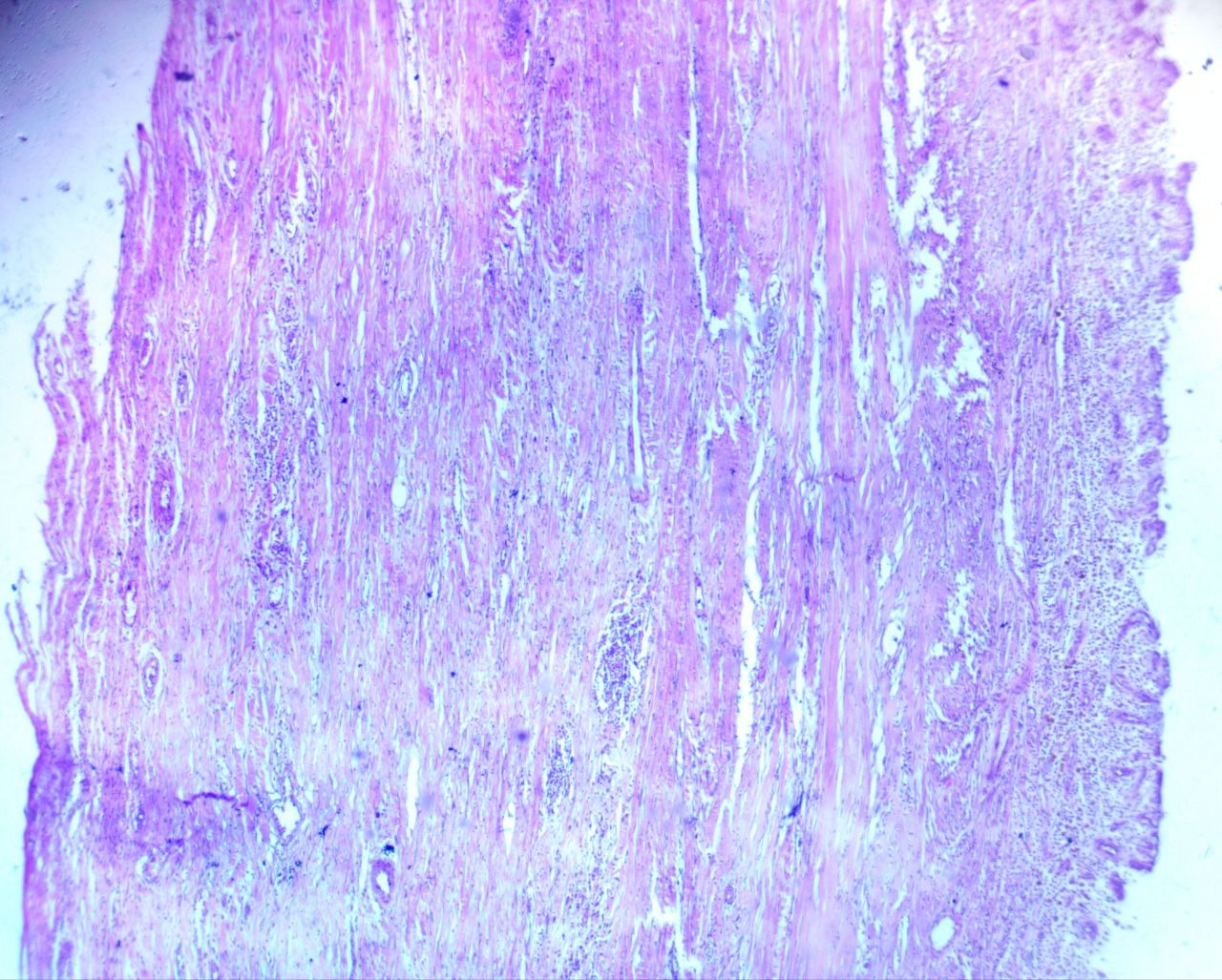

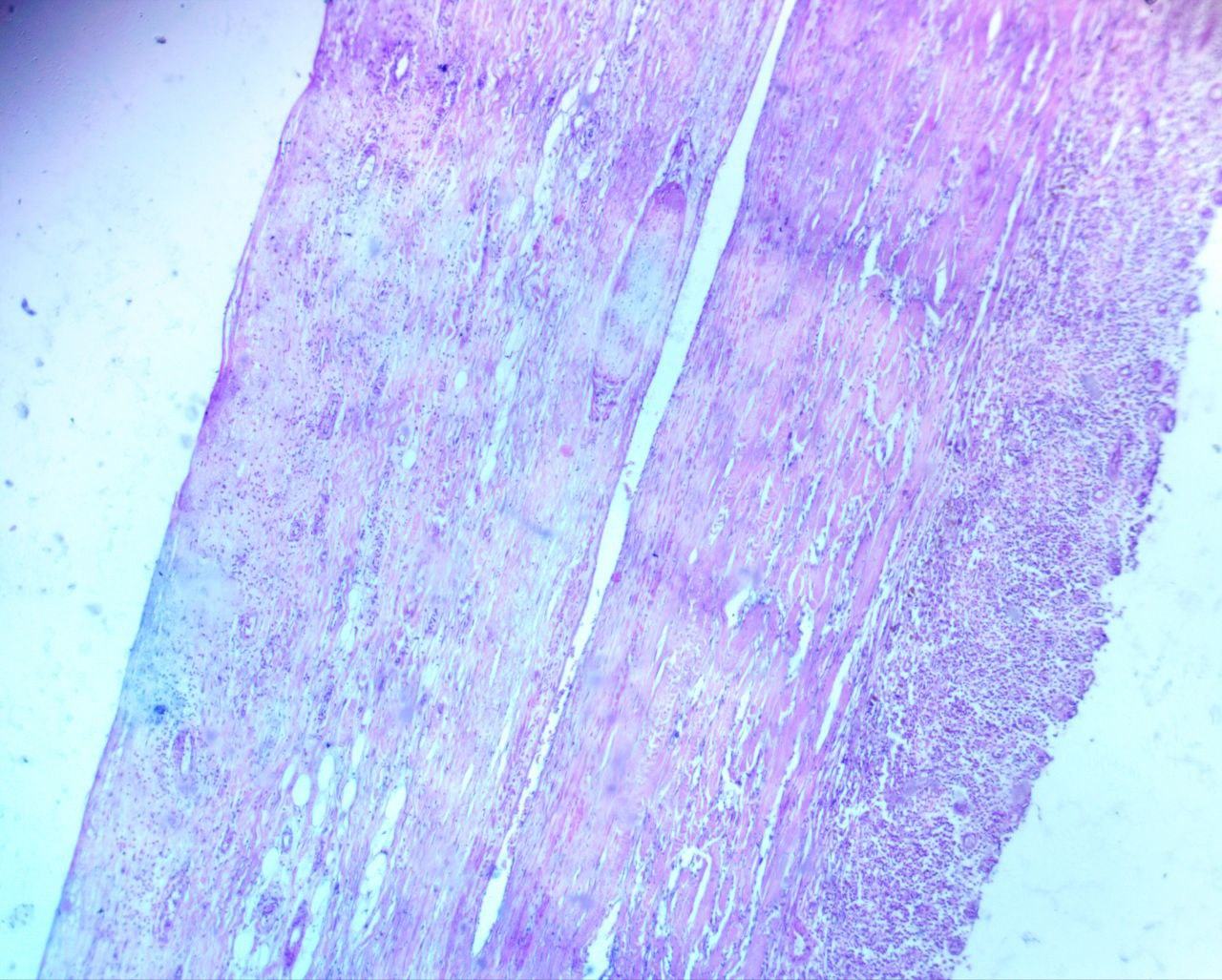

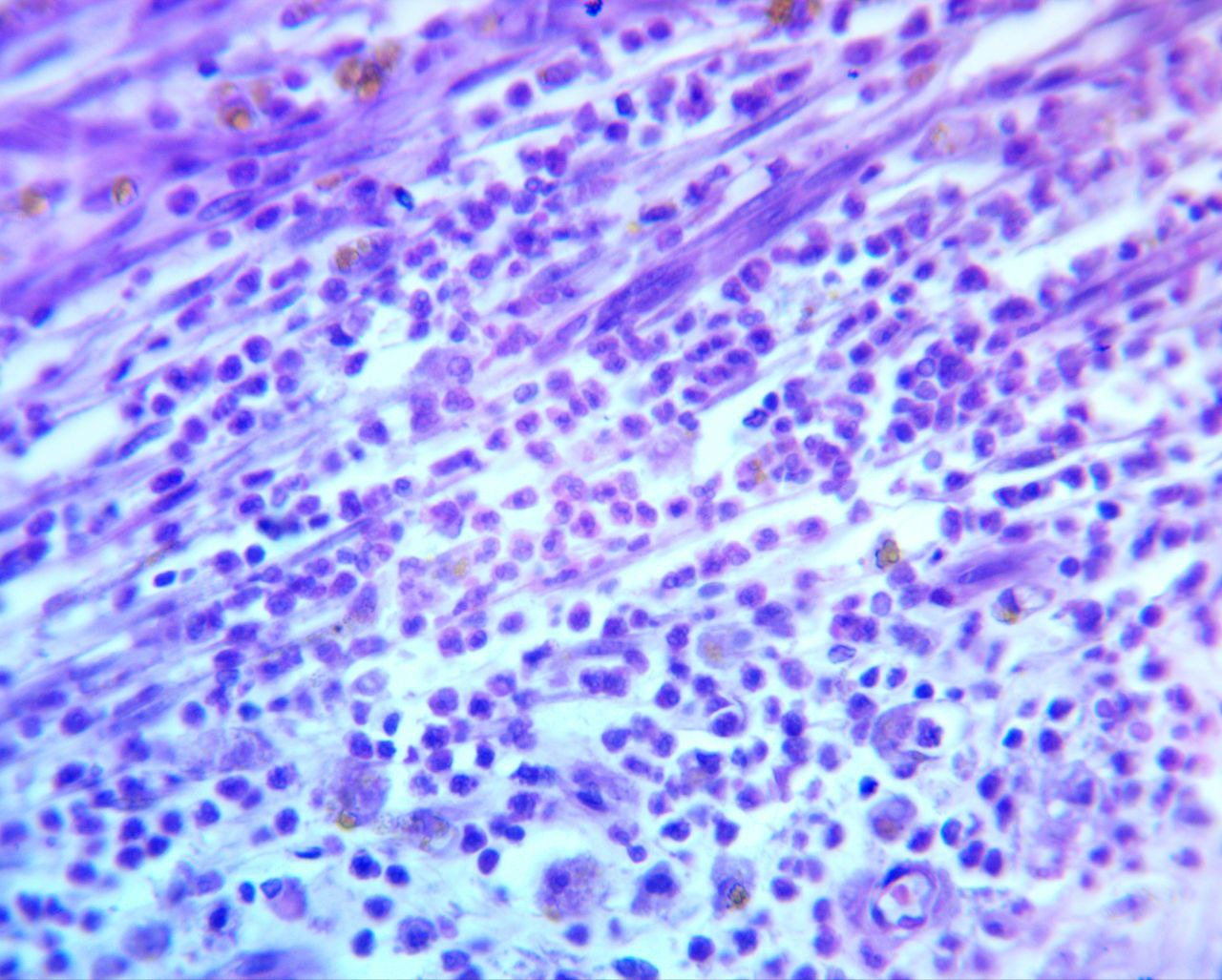

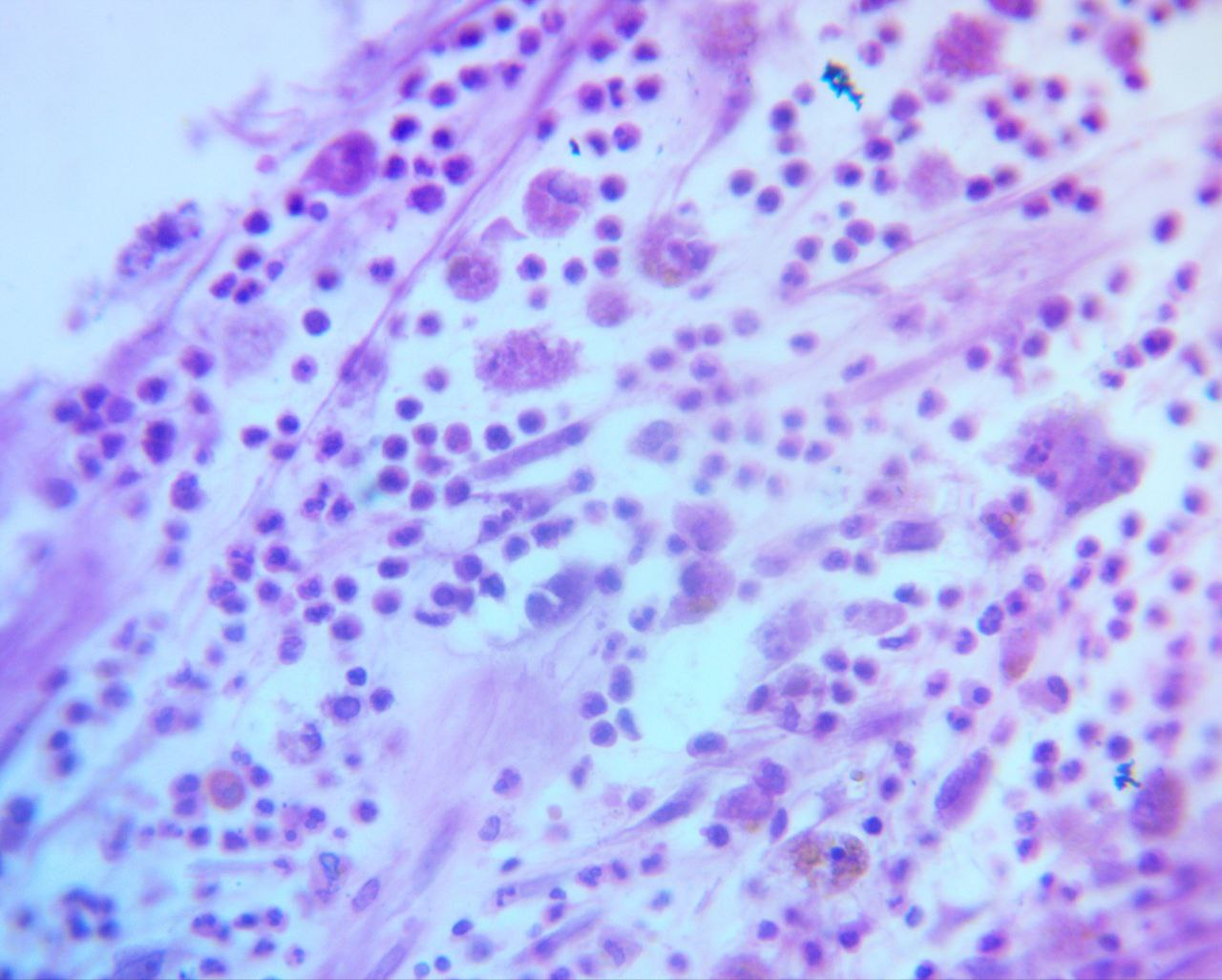

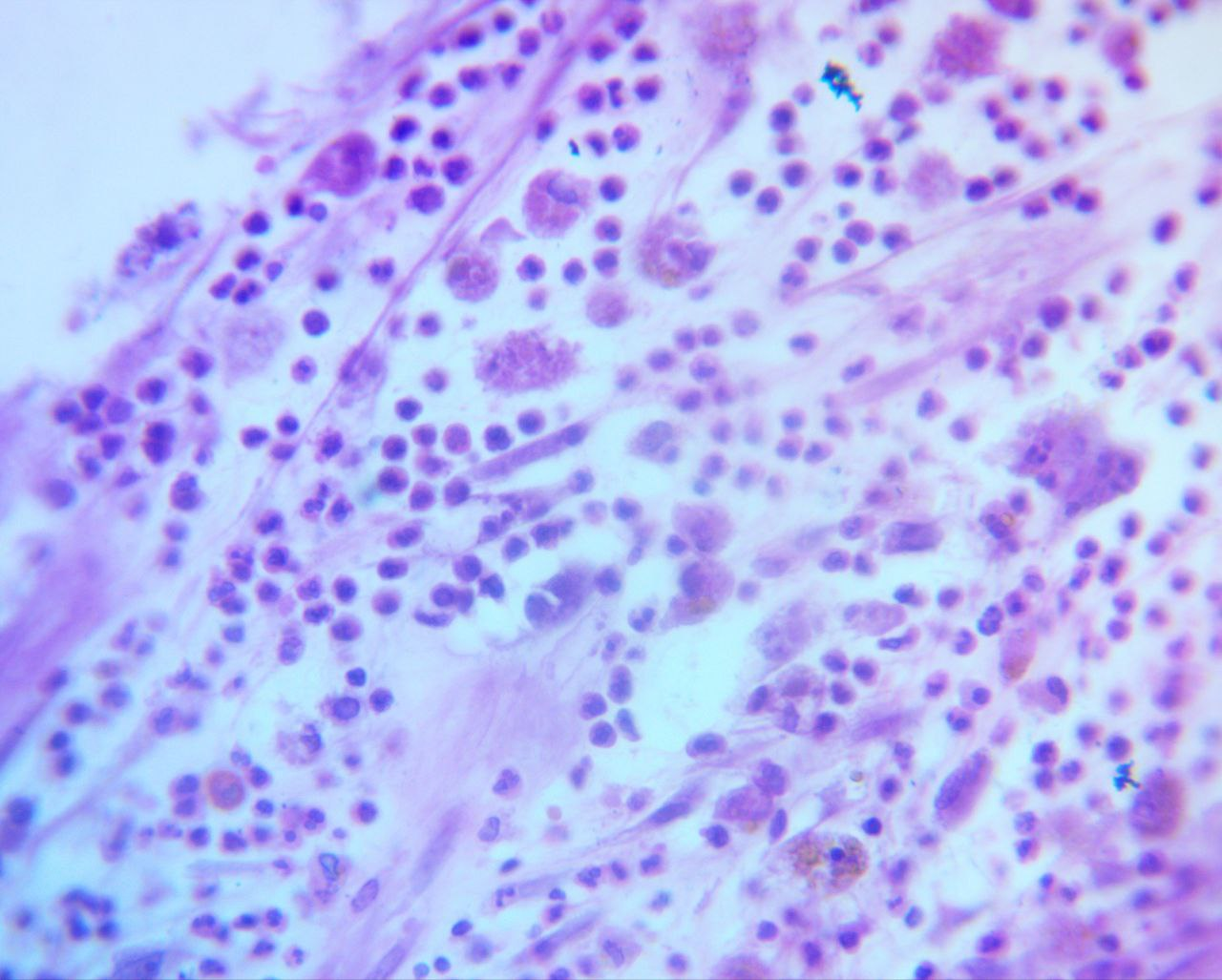

Supplement: Supplementary file 1 — Additional file 1. Adjacent to Figure 8 and 9. [file 13256_2024_4503_MOESM1_ESM.docx]
